# Social disconnection correlates of a “Wish to Die” among a large community-dwelling cohort of older adults

**DOI:** 10.3389/fpubh.2024.1436218

**Published:** 2024-08-21

**Authors:** Mark Ward, Robert Briggs, Rose Anne Kenny

**Affiliations:** ^1^The Irish Longitudinal Study on Ageing (TILDA), Trinity College Dublin, Dublin, Ireland; ^2^Department of Medical Gerontology, Trinity College Dublin, Dublin, Ireland; ^3^Mercers Institute for Successful Ageing, St James's Hospital, Dublin, Ireland

**Keywords:** social disconnection, death ideation, wish to die, older adults, loneliness, social isolation

## Abstract

**Introduction:**

Social disconnection and deaths by suicide among older adults are both important public health concerns, particularly in the context of ageing populations. The association between death ideation and behaviours, and social disconnection is well established and both functional and structural social relationships have been identified as predictive of suicide-related thoughts and behaviours. The “Wish to Die” (WTD) involves thoughts of or wishes for one’s own death or that one would be better off dead is a commonly used indicator to capture death ideation. It has been shown to be as predictive as active ideation of future suicide attempt.

**Methods:**

Data were from a large cohort of community-dwelling older adults aged 50+, The Irish Longitudinal Study on Ageing (TILDA). Cross-sectional analyses of the association between numerous markers of social disconnection (loneliness, social isolation, living alone, marital status, social participation, volunteering, and attending religious service) and WTD were conducted.

**Results:**

Multiple markers of social disconnection were associated with a “wish to die”. However, loneliness was the strongest risk factor while attendance of religious services was an important protective behaviour.

**Discussion:**

There is a strong association between social disconnection and a WTD among older adults. There is also a strong association between depression and a WTD, while attending religious services or similarly prosocial settings may protect older adults from experiencing negative thoughts about dying.

## Introduction

The earliest theory of suicide that emphasised the importance of social disconnection was developed by Emile Durkheim in 1897 (1). Since then, social disconnection has consistently been shown to correlate with both death ideation and suicidal thoughts and behaviours (2, 3). Deaths by suicide and social disconnection among older adults are both increasingly serious public health concerns, particularly in the context of ageing populations. Suicide rates among older adults are increasing in Ireland and elsewhere, with significant increases among women and those aged 55 to 64 years in particular (4). Higher rates of death ideation are also observed among older adults compared to younger people (5) and subsequent suicide attempts among older adults are more likely to be fatal (6). Death ideation is therefore an important clinical marker for future suicidal behaviour (7) and passive suicidal ideation is a core element of the definition of suicide attempt used by the National Institute of Mental Health (8).

The “Wish to Die” (WTD) is a commonly used indicator in observational studies to operationalise death ideation and has been shown to be as predictive of future suicide attempts as active suicide ideation (9). A WTD involves an individual’s thoughts of one’s own death, that one would be better off dead, or wishing for one’s death and it is a clinical marker for future suicidal behaviour (9). In Europe, 12% of older adults reported current WTD (10) and 3% of Irish adults report WTD in the previous month (11). A WTD, depression and other measures of psychologically distress are established risk factors for suicidal ideation (9) and behaviours (12). The WTD among community-dwelling older people does not tend to persist over time and instead tends to be transient. Furthermore, the trajectory of WTD tends to mirror that of depressive symptoms and loneliness within individuals which again emphasises the strength of importance of the association between these conditions (11).

Social disconnection is associated with an increased likelihood of death ideation (13, 14), with both social isolation and loneliness strongly related to both suicide ideation and attempts with the risk of both increasing with the level of disconnection (15). Social disconnection is often narrowly defined in terms of either loneliness or social isolation. Loneliness is the subjective assessment of an individual’s satisfaction with the quality of their social relationships (16) while social isolation on the other hand, is an objective measure or count of an individual’s social contacts (17). A systematic review of the prospective association between loneliness specifically and death ideation and behaviours found that loneliness was predictive and that this association was mediated by depression (18). Likewise, social isolation has been found to be predictive of suicide risk particularly among older adults and adolescents (18). The evidence therefore is clear that there is an association between suicidal thoughts and behaviours and both subjective (loneliness) and objective (isolation) measures of social disconnection and this supports calls for both to be included in risk assessments for suicide (2).

Beyond loneliness and social isolation, Bernier et al. (19) found that after controlling for depression and other factors, a number of other markers of social disconnection were associated with WTD, including marital status and social participation. Similarly, a review by Calati et al. reported that suicidal outcomes were associated with living alone while being married was a protective factor (2). Finally, a meta-analysis by Chang et al. (20) examined the association between suicidal ideation and both structural (for example, marital status, household composition) and functional social relationships, with functional social relationships defined as perceived social connections, including loneliness. Among the 31 studies with 203,152 participants included in their analysis, they found that functional measures were more predictive than structural ones with mistreatment by others and loneliness the most important factors. These findings provide support for the interpersonal theory of suicide, first proposed by Joiner (21, 22) and further developed by Van Orden et al. (6), which states that the WTD is a response to thwarted belongingness (unmet need for social connectedness) and perceived burdensomeness (on others).

Another important consideration, particularly in the Irish context is the central role that religion, and Roman Catholicism in particular, plays in Ireland (23). In 2022, over 80% of adults aged 60 and older identified as Roman Catholic although the number of younger adults doing so has been decreasing (24). Given the centrality of religion to so many aspects of social life in Ireland, it is essential that we account for religiousness when examining suicidal behaviours, and indeed, social disconnection in an Irish context. There are two potential reasons for this. Firstly, religion is associated with lower loneliness (25) and depression (26). This is most likely due to the opportunity for social connection associated with attending religious services and also the spiritual support from other church members. Secondly, the comfort received from religion has been identified as an adaptive response that protects against death ideation (27). Additionally, the Catholic church, in its teaching and practice of Canon law in Ireland contributed greatly to a heightened level of taboo and stigma associated with suicide. In practical terms, this meant that until the 1980s, the Catholic Church would not conduct funeral services for individuals who took their own lives, and they could not be buried in a Catholic cemetery (28). This heightened stigma means that older Catholics may be less likely to consider suicidal thoughts or at least less likely to report experiencing these feelings.

In this study, we examined the association between WTD and a number of both structural and functional indicators of social relationships. Our aims in doing so are twofold. Firstly, we wished to describe the association between social disconnection and a WTD among a large nationally representative cohort of community-dwelling older adults. Secondly, we wanted to compare the strength of the association between WTD and a variety of commonly used structural and functional measures of social disconnection. In doing so we may identify and subsequently prioritise specific domains of social connectedness. These findings can then inform coping strategies and interventions that manipulate social connectedness as a method to prevent the progression of passive suicidal thoughts to active ones.

## Methodology

Data were from The Irish Longitudinal Study on Ageing (TILDA). TILDA is a sister study of the Health and Retirement Study (HRS). It is a prospective, nationally representative study of community-dwelling adults aged ≥50 years resident in the Republic of Ireland. A detailed description of the methodologies employed by TILDA are available elsewhere (29–32). Briefly, participants were selected via multi-stage stratified random sampling which consisted of the selection of 640 geographical areas, stratified by socioeconomic characteristics, followed by the random selection of forty households within each of these areas. The sampling frame was the Irish GeoDirectory which provides a list of all residential addresses. 8,175 Computer Assisted Personal Interviews (CAPI) were conducted at baseline were completed with a response rate of 62%. 85% (*n* = 6,915) of these respondents also returned completed Self-Completion Questionnaires (SCQs).

## Dependent variable

A single item question on WTD was administered via CAPI questionnaire. The question asked: “In the last month, have you felt that you would rather be dead?” The response categories were (0 = No such feelings; 1 = Any mention of suicidal feelings or wishing to be dead).

## Independent variables

*Loneliness* was measured using the five-item version of the of the University of California Los Angeles (UCLA) loneliness scale (33). This well validated scale consists of five items: How often do you feel you lack companionship? How often do you feel left out? How often do you feel isolated from others? How often do you feel in tune with the people around you? How often do you feel lonely? The response options are hardly ever or never = 0, some of the time = 1, often = 2. Responses to the five items are then summed, resulting in scores ranging from 0 (not lonely) to 10 (extremely lonely). *Social isolation* was measured using the Berkman-Syme Social Network Index (SNI) (17). The SNI includes four types of social connection: (1) marital status; (2) close ties with children, relatives, and friends; (3) membership of a church group, and (4) membership of voluntary organisations. A score of 0–1 indicates “most isolated”, with a score of 4 indicating “most integrated”. The other indicators included were whether participants lived alone or not (yes/no), and their marital status (yes, married or cohabiting / not married). Social participation was measured in the CAPI by asking whether participants “participate in any groups such as a sports or social group or club, a church connected group, a self-help or charitable body or other community group or a day care centre?” As part of the SCQ, participants were asked if they had done voluntary work in the previous year (yes/no). The frequency (never, monthly, weekly) that participants attended religious services was recorded as part of the CAPI. Finally, depression was also measured during CAPI using the Center for Epidemiological Studies Depression scale (CES-D) (34). This commonly used and previously validated 20-item scale measures the frequency that respondents experienced a variety of depressive symptoms within the past week with higher scores indicating increased depressive symptomology (35). CES-D scores ranged from 0 to 60 and the scale had good internal reliability (α = 0.88). A CES-D score of 16 was used to indicate clinically significant depressive symptoms (36) and the resultant binary variable (not depressed / depressed) was used in all analyses.

## Statistical approach

**Figure 1:**
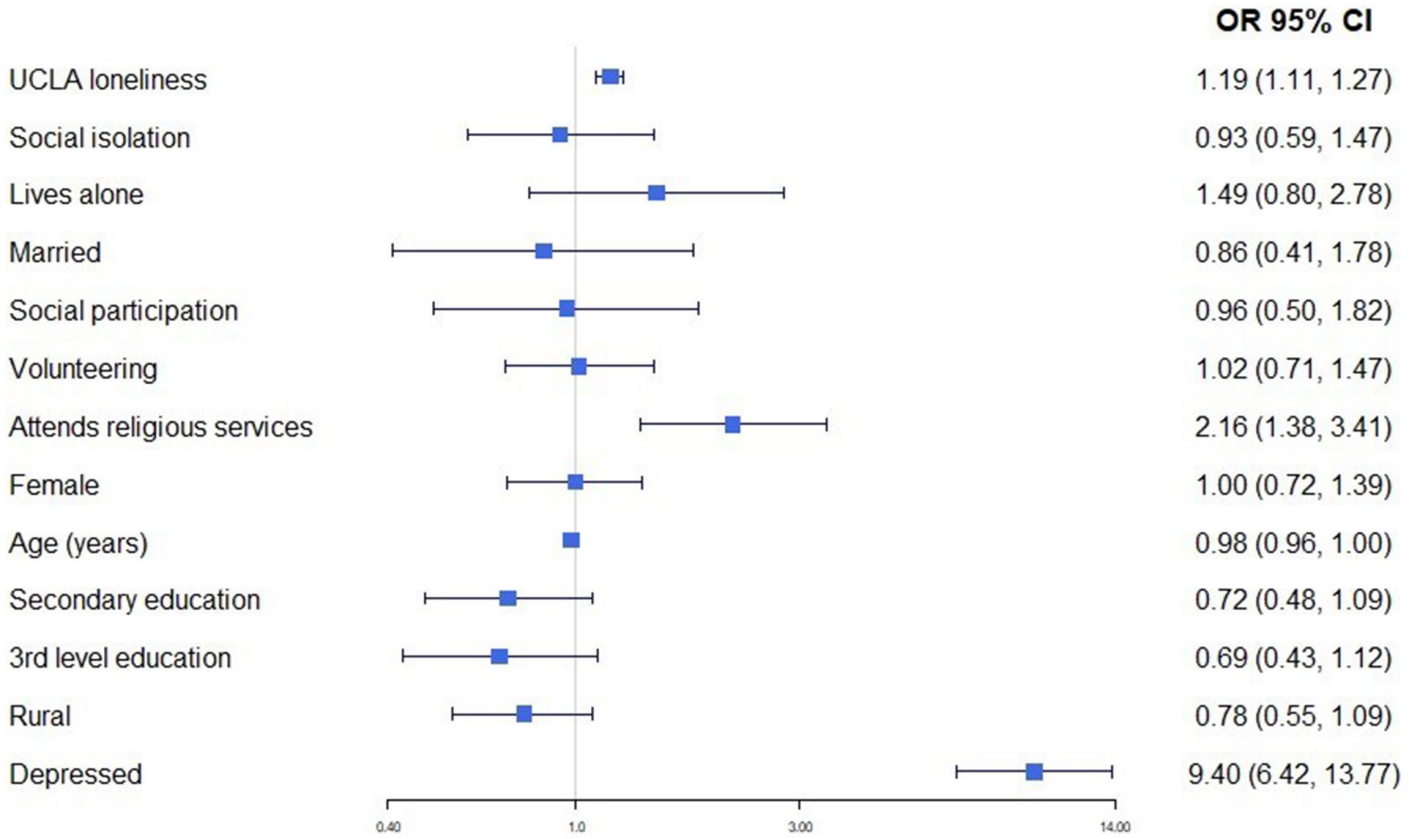
Results of binary logistic regressions to estimate the association between social disconnection indicators and WTD, controlling for socio-demographic characteristics (O.R, 95%CI).

We conducted cross-sectional analyses of data from wave 1 of TILDA. As noted above, data for some of the key variables were collected as part of the SCQ. To better ensure comparability across the different statistical tests, our analytic sample included participants who completed the SCQ. T-tests were used to compare means and chi-square tests to test the association between categorical variables in bivariate analyses. Binary logistic regression models were estimated for the main analyses where the dichotomous WTD indicator was the dependent variable. The results of these regressions were reported as odds ratios and 95% confidence intervals were reported for all point and model estimates. Inverse probability weights were applied in all analyses. These weights were estimated by comparing the age, sex, educational attainment, marital status, and geography of participants to their distribution in the Irish Census of population data. The survey weights also adjusted the data for clustering due to the multi-stage sampling design. Analyses were conducted using Stata/MP 15.1 (37). The forest plot at Figure 1 was created using R 4.3.1 (38).

## Sensitivity analysis

Because the UCLA loneliness items were administered in the SCQ, participants who completed the CAPI but not the SCQ were excluded from our analyses. This was to allow us to directly compare the strength of associations between the social disconnection variables and WTD, among the same participants. To assess whether there may be systematic, non-random, differences on key variables between those who did and those who did not complete the SCQ, we repeated the analyses with all participants, including those who completed the CAPI but not the SCQ included. The results of these analyses are presented in Supplementary Tables S1, S2. Survey weights designed specifically for CAPI participation were applied for these additional analyses.

## Results

The socio-demographic characteristics of participants are provided in Table 1 and disaggregated by WTD (yes/no). The prevalence of depression (10%) and WTD (3.6%) are also shown. A significantly higher percentage of participants who completed only primary level education reported a WTD. Older adults who lived in urban areas and those with clinically significant depressive symptomology were also more likely to report a WTD in the previous month.

**Table 1:** Socio demographic characteristics of older adults expressing a WTD (*N* = 6,911).

	All	No WTD	Yes WTD	Sig. Test
	% (95% CI)	% (95% CI)	% (95% CI)	
No WTD	96.4 (95.9,96.8)			
Yes WTD	3.6 (3.2,4.1)			
Male	47.9 (46.9,48.9)	96.7 (95.9,97.3)	3.3 (2.7,4.1)	χ^2^(1) = 1.10, *p* = 0.345
Female	52.1 (51.1,53.1)	96.2 (95.4,96.9)	3.8 (3.1,4.6)	
Mean age	62.8 (61.1,64.5)	64.1 (63.7,64.4)	62.8 (61.1,64.5)	t = −1.5, *p* = 0.135
Primary education	38.2 (36.6,39.9)	95.3 (94.1,96.2)	4.7 (3.8,5.9)	χ^2^(2) = 15.96, *p* < 0.001
Secondary education	43.2 (41.8,44.6)	97.1 (96.4,97.7)	2.9 (2.3,3.6)	
3rd level education	18.6 (17.5,19.7)	97.1 (96.2,97.8)	2.9 (2.2,3.8)	
Urban	50.3 (46.5,54.1)	95.4 (94.5,96.1)	4.6 (3.9,5.5)	χ^2^(1) = 21.42, *p* < 0.001
Rural	49.7 (45.9,53.5)	97.5 (96.8,98.0)	2.5 (2.0,3.2)	
Not depressed	90.0 (89.1,90.9)	98.5 (98.1,98.8)	1.5 (1.2,1.9)	χ^2^(1) = 674.2, *p* < 0.001
Depressed	10.0 (9.1,10.9)	79.3 (75.7,82.5)	20.7 (17.5,24.3)	

In Table 2, we report the prevalence of a WTD by each of the indicators of social disconnection described above. Older adults who reported a WTD reported higher levels of loneliness and social isolation. They were also significantly more likely to live alone and be unmarried. They also reported less social participation, never volunteer, and did not regularly attend religious services. This latter group which included older adults with or without a religion who never attended church reported the highest prevalence of WTD at 8.7%. This is 2.4 times the overall proportion of 3.6%. Overall, 88.6% of participants were Roman Catholic with Anglican the next largest group at 3.4%.

**Table 2:** Prevalence of multiple indicators of social disconnection among older adults expressing a WTD (*N* = 6,911).

	All	No WTD	Yes WTD	Sig. Test
	% (95% CI)	% (95% CI)	% (95% CI)	
Mean UCLA loneliness	2.0 (1.9,2.0)	1.9 (1.8,2.0)	4.4 (4.0,4.9)	t = 11.73, *p* < 0.001
Mean SNI	2.8 (2.7,2.8)	2.8 (2.8,2.8)	2.3 (2.1,2.4)	t = −8.9, *p* < 0.001
Lives alone	23.0 (21.7,24.2)	94.5 (93.0,95.7)	5.5 (4.3,7.0)	χ^2^(1) = 22.0, *p* < 0.001
Lives with others	77.0 (75.8,78.3)	97.0 (96.4,97.5)	3.0 (2.5,3.6)	
Not married	32.2 (30.7,33.6)	94.8 (93.6,95.8)	5.2 (4.2,6.4)	χ^2^(1) = 23.6, *p* < 0.001
Married	67.8 (66.4,69.3)	97.2 (96.6,97.7)	2.8 (2.3,3.4)	
No social participation	54.1 (52.6,55.6)	95.5 (94.6,96.2)	4.5 (3.8,5.4)	χ^2^(1) = 21.0, *p* < 0.001
Yes social participation	45.9 (44.4,47.4)	97.5 (96.9,98.0)	2.5 (2.0,3.1)	
Volunteer	49.1 (47.6,50.5)	97.5 (96.9,98.0)	2.5 (2.0,3.1)	χ^2^(1) = 23.2, *p* < 0.001
No volunteer	50.9 (49.5,52.4)	95.3 (94.4,96.1)	4.7 (3.9,5.6)	
Frequency attends church				
Never	15.6 (14.3,16.9)	91.3 (89.2,93.0)	8.7 (7.0,10.8)	χ^2^(2) = 100.8, *p* < 0.001
Monthly	25.8 (24.6,27.1)	96.8 (95.7,97.6)	3.2 (2.4,4.3)	
Weekly	58.6 (56.9,60.3)	97.6 (97.0,98.1)	2.4 (1.9,3.0)	

The results of a series of binary logistic regressions to estimate the strength of the association between a WTD and our seven social disconnection indicators, while adjusting our estimates for socio-demographic characteristics and depression, are presented in Table 3. While there was a strong independent association between each indicator and the likelihood of reporting a WTD in the unadjusted models, the association with social participation and volunteering was no longer significant when the models were adjusted for covariates. An increase in one-point on the UCLA loneliness scale was associated with an increased odds ratio (O.R. = 1.21, 95% CI:1.13,1.39) of reporting a WTD. Similarly, the likelihood of reporting a WTD increased significantly with each extra point on the Berkman-Syme Social Network Index (O.R. = 0.68, 95% CI:0.57,0.82). In order of the strength of association the following measures of social disconnection were also associated with an increased likelihood of WTD: attending church (O.R. = 2.50, 95% CI:1.76,3.55); living alone (O.R. = 1.66, 95% CI:1.18,2.33); unmarried (O.R. = 1.48, 95% CI:1.05,2.07).

**Table 3:** Results of binary logistic regressions to estimate the association between social disconnection indicators and WTD, controlling for socio-demographic characteristics (*N* = 6,911).

	UCLA loneliness	Social isolation	Lives alone	Married	Social participation	Volunteers	Attends church
	O.R. (95% CI)	O.R. (95% CI)	O.R. (95% CI)	O.R. (95% CI)	O.R. (95% CI)	O.R. (95% CI)	O.R. (95% CI)
Unadjusted	1.44 (1.37,1.52)***	0.51 (0.44,0.60)***	1.88 (1.38,2.56)***	1.86 (1.38,2.52)***	1.87 (1.39,2.52)***	1.93 (1.42,2.63)***	3.55 (2.63,4.79)***
Adjusted	1.21 (1.13,1.29)***	0.68 (0.57,0.82)***	1.66 (1.18,2.33)**	1.48 (1.05,2.07)*	1.35 (0.97,1.87)	1.29 (0.93,1.79)	2.50 (1.76,3.55)***
Female	0.89 (0.65,1.23)	0.89 (0.66,1.21)	0.88 (0.65,1.20)	0.85 (0.62,1.16)	0.86 (0.63,1.16)	0.84 (0.61,1.15)	0.96 (0.70,1.31)
Age	0.98 (0.96,1.00)	0.99 (0.98,1.01)	0.99 (0.97,1.01)	0.99 (0.97,1.01)	0.99 (0.97,1.01)	0.99 (0.97,1.01)	1.00 (0.98,1.02)
Primary education Ref.							
Secondary	0.69 (0.47,1.01)	0.76 (0.54,1.09)	0.71 (0.50,1.01)	0.72 (0.51,1.01)	0.73 (0.51,1.04)	0.71 (0.50,1.02)	0.75 (0.53,1.07)
3rd level	0.70 (0.45,1.09)	0.79 (0.51,1.20)	0.68 (0.45,1.04)	0.69 (0.46,1.05)	0.74 (0.49,1.14)	0.73 (0.47,1.14)	0.68 (0.44,1.04)
Rural	0.65 (0.47,0.90)**	0.65 (0.48,0.90)**	0.60 (0.44,0.83)**	0.61 (0.44,0.83)**	0.60 (0.44,0.83)**	0.63 (0.45,0.87)**	0.70 (0.51,0.96)*
Depressed	10.20 (6.98,14.91)***	14.01 (10.10,19.44)***	15.27 (11.05,21.10)***	15.28 (10.98,21.26)***	15.67 (11.33,21.68)***	16.18 (11.60,22.58)***	14.86 (10.73,20.58)***

*p<0.05; **p<0.01; ***p<0.001.

Among the covariates, living in a rural area was associated with a decreased likelihood of a WTD in each model. This decrease ranged from 0.3 in the church attendance model to 0.4 in the model of living alone. The association between WTD and depression was consistently high in each model.

In the final analysis, each social disconnection indicator was included in the one binary logistic regression so to identify the most important predictors while controlling for other measures. The resultant estimates are presented in Figure 1. The strongest association was between depression and WTD (O.R. = 9.40, 95% CI:6.42,13.77) followed by attending church (O.R. = 2.16, 95% CI:1.38,3.41) and UCLA loneliness scores (O.R. = 1.19, 95% CI:1.11,1.27).

The results of the sensitivity analyses (N = 8,171) in which we re-estimated the binary logistic regression analyses with all participants, including those who completed the CAPI but not the SCQ, are provided in Supplementary Tables S1, S2. There was virtually no difference between these additional analyses and the main analyses in the size or direction of the observed estimates. These results show that there were no systematic, non-random, differences on key variables between participants who did and those who did not complete the SCQ.

## Discussion

Using data from a large, nationally representative cohort of community-dwelling older adults, this study provided evidence of a strong association between a WTD and both structural and functional measures of social disconnection commonly included in observational studies of older adults. We have also shown the particularly strong association between both loneliness and depression and a WTD, as well as the protective role that regularly attending religious services may play. While the prevalence of WTD among this cohort of older adults have been previously reported (12) along with its association with depression and loneliness (13), this study extends that work by assessing the utility of a range of commonly used measures of social disconnection.

It is clear from these and other findings that a WTD is strongly related to both depression (12, 13, 35, 36) and social disconnection (18, 19), with loneliness a key indicator of the latter. Importantly, social isolation and loneliness are correlated not only with suicidal ideation but also behaviours (9, 10). While previous research has identified both loneliness and social isolation (18) as risk factors for suicidal ideation, our findings suggest that loneliness—the subjective assessment of the quality of an individuals’ social relationships (16), as opposed to a count of their social contacts (social isolation) (17)—is the more important of the two. The association between WTD and loneliness remained strong even after controlling for depression and other markers of social disconnection. While we did find evidence of an association between WTD and both structural and functional social relationships. Similar to the review by Chang et al. (20), the functional measure of loneliness was the strongest predictor. This finding provides further support for the importance of thwarted belongness emphasised in the interpersonal theory of suicide (6, 22, 23). There is growing evidence that both depression and loneliness are amenable to intervention (39, 40) and these interventions may therefore also help protect against feelings of wishing to die.

While it is not clear whether it is the spiritual or the social aspect of attending church that provides the mechanism, our finding of the strong protective role of religious attendance also suggests a potential coping strategy that that may be amenable to older adults. If it is the sociality rather than the spirituality of attending church regularly that is more important, then social activities not only those associated with religious practice may be a more attractive proposition, particularly if the current trend of increasing secularity continue. While attendance at religious attendance is provided here as an example of a beneficial prosocial activity, there are of course numerous examples of secular prosocial activities. Given the strength of the association between loneliness and WTD, existing interventions to address the former may have the additional benefit of protecting against death ideation. There are currently more than 316 interventions addressing loneliness and many of these consist of efforts to connect people through group social activities (41). There is also evidence that cognitive behaviour therapy interventions targeting depression and anxiety may also reduce the burden of loneliness (42). As well as interventions at the individual level we also need to better understand the potential role of macro-level factors that promote or inhibit social well-being. A recent proposal promoting prosociality as a novel method to improve population health offers an example of one such public health strategy to improve birth individual and societal health (43).

The findings presented here are important for a number of reasons, particularly increasing suicide rates among older adults (4) and the fact that death ideation is an important clinical marker for future suicidal behaviour (7). Furthermore, it is important to view these findings in the context of our emerging from the recent COVID-19 pandemic which led to significant increases in important and inter-related predictors of WTD identified here—depression and social disconnection (44). Indeed, the pandemic also saw religious attendance severely curtailed for long periods (45). While we do not have information on WTD among this cohort post-pandemic, it is fair to suspect that the combination of an increase in risk via depression and social disconnection coupled with a decrease in the protective role of religious attendance, may have resulted in a greater number of older adults experiencing a WTD. Finally, as noted previously by Briggs (12), the Dying with Dignity Bill 2020, that would legalise assisted dying for those with terminal illnesses is still being considered by legislators and other stakeholders in Ireland. The data presented here provides important information on a cohort of older adults who have expressed a WTD.

We have noted that suicide has been highly stigmatised in Ireland, particularly through teachings of the Roman Catholic Church. As the vast majority (88.6%) of participants were Roman Catholic and this stigma is likely more heightened among these older adults. Furthermore, participants experience of a WTD was only asked for the last month and individuals who have had WTD outside this timeframe are therefore excluded. For these reasons a limitation of this study is that it is possible that the prevalence of WTD in this sample is an under-estimation of the true population value. We limited our analyses to cross-sectional data to provide a framework within which to establish the association between social disconnection and WTD and to compare the utility of different measures of both structural and functional social disconnection. We therefore cannot speak to the directionality of the associations we have observed nor the development and trajectory of a WTD or social disconnection. Future research will examine the trajectory and longitudinal associations between WTD and social disconnection and will shed further light on mechanisms that may explain the associations reported here. A key strength of our study is the use of a large population representative sample of older adults, and the inclusion of multiple measures of disconnection which we hope helps prioritise loneliness as the vital indicator of social disconnection.

## Conclusion

An important objective of our examining different indicators of social disconnection was to identify specific domains of social connectedness that might be prioritised to screen or prevent WTD among older adults. While each indicator was to lesser or greater extent associated with an increased risk of WTD, loneliness and attending religious services dominated, even after controlling for depression. This suggests that enabling engagement in prosocial settings, religious or not, may protect older adults from experiencing negative thoughts about dying. These findings can inform effective clinical and community-based interventions by which the train of thoughts leading to active suicidal ideation can be stopped before individuals progress to active ideation and suicidal behaviour.

## Data availability statement

Publicly available datasets were analyzed in this study. This data can be found here: https://www.ucd.ie/issda/data/tilda/.

## Ethics statement

The studies involving humans were approved by ethical approval for the TILDA study is granted by the Faculty of Health Sciences Research Ethics Committee at Trinity College Dublin REC Ref: 190407. The studies were conducted in accordance with the local legislation and institutional requirements. The participants provided their written informed consent to participate in this study.

## Author contributions

MW: Conceptualization, Formal analysis, Funding acquisition, Investigation, Methodology, Project administration, Writing – original draft, Writing – review & editing. RB: Conceptualization, Writing – review & editing, Funding acquisition. RK: Conceptualization, Funding acquisition, Resources, Supervision, Writing – review & editing.

## References

[1] Durkheim E. Suicide: A study in sociology. London: Routledge (1897).

[2] Calati R Ferrari C Brittner M Oasi O Olié E Carvalho AF. Suicidal thoughts and behaviors and social isolation: a narrative review of the literature. J Affect Disord. (2019) 245:653–67. doi: 10.1016/j.jad.2018.11.022, PMID: 30445391

[3] Cheung G Edwards S Sundram F. Death wishes among older people assessed for home support and long-term aged residential care: death wishes among older people. Int J Geriatr Psychiatry. (2017) 32:1371–80. doi: 10.1002/gps.4624, PMID: 27859762

[4] Griffin E McTernan N Wrigley C Nicholson S Arensman E Williamson E. *National Self-Harm Registry Ireland – Annual Report 2018*, pp. 166–169 (2019).

[5] Fiske A Wetherell JL Gatz M. Depression in older adults. Annu Rev Clin Psychol. (2009) 5:363–89. doi: 10.1146/annurev.clinpsy.032408.153621, PMID: 19327033 PMC2852580

[6] van Orden KA Witte TK Cukrowicz KC Braithwaite SR Selby EA Joiner TE. The interpersonal theory of suicide. Psychol Rev. (2010) 117:575–600. doi: 10.1037/a0018697, PMID: 20438238 PMC3130348

[7] Baca-Garcia E Perez-Rodriguez MM Oquendo MA Keyes KM Hasin DS Grant BF. Estimating risk for suicide attempt: are we asking the right questions? J Affect Disord. (2011) 134:327–32. doi: 10.1016/j.jad.2011.06.026 21784532 PMC3172880

[8] O'Carroll PW Berman AL Maris RW Moscicki EK Tanney BL Silverman MM. Beyond the tower of babel: a nomenclature for suicidology. Suicide Life Threat Behav. (1996) 26:237–52. doi: 10.1111/j.1943-278X.1996.tb00609.x, PMID: 8897663

[9] Harmer B Lee S Duong T Saadabadi A. Suicide Ideation StatPearls Publishing (2021).

[10] Stolz E Fux B Mayerl H Rásky É Freidl W. Passive suicide ideation among older adults in Europe: a multilevel regression analysis of individual and societal determinants in 12 countries (SHARE). J Gerontol B Psychol Sci Soc Sci. (2016) 71:947–58. doi: 10.1093/geronb/gbw041, PMID: 27048569 PMC4982389

[11] Briggs R Ward M Kenny RA. The ‘wish to die’ in later life: prevalence, longitudinal course and mortality. Data from TILDA. Age Ageing. (2021) 50:1321–8. doi: 10.1093/ageing/afab010, PMID: 33570600 PMC7929464

[12] Conwell Y Van Orden K Caine ED. Suicide in older adults. Psychiatr Clin N Am. (2011) 34:451–68. doi: 10.1016/j.psc.2011.02.002, PMID: 21536168 PMC3107573

[13] Briggs R Tobin K Kenny RA Kennelly SP. What is the prevalence of untreated depression and death ideation in older people? Data from the Irish longitudinal study on aging. Int Psychogeriatr. (2018) 30:1393–401. doi: 10.1017/S104161021700299X, PMID: 29335038

[14] Lutz J van Orden K Bruce ML Conwell Y Members of the NIMH Workshop on Social Disconnection in Late Life Suicide. Social disconnection in late life suicide: an NIMH workshop on state of the research in identifying mechanisms, treatment targets, and interventions. Am J Geriatr Psychiatry. (2021) 29:731–44. doi: 10.1016/j.jagp.2021.01.137, PMID: 33622593 PMC8286287

[15] Stravynski A Boyer R. Loneliness in relation to suicide ideation and parasuicide: a population-wide study. Suicide Life Threat Behav. (2001) 31:32–40. doi: 10.1521/suli.31.1.32.21312 11326767

[16] Perlman D Peplau LA. Toward a social psychology of loneliness In: Duck SW Gilmour R, editors. Personal relationships in disorder. London: Academic Press (1981). 31–56.

[17] Berkman LF Syme SL. Social networks, host resistance, and mortality: a nine-year follow-up study of Alameda County residents. Am J Epidemiol. (1979) 109:186–204. doi: 10.1093/oxfordjournals.aje.a112674, PMID: 425958

[18] McClelland H Evans JJ Nowland R Ferguson E O'Connor RC. Loneliness as a predictor of suicidal ideation and behaviour: a systematic review and meta-analysis of prospective studies. J Affect Disord. (2020) 274:880–96. doi: 10.1016/j.jad.2020.05.004, PMID: 32664029

[19] Bernier S Lapierre S Desjardins S. Social interactions among older adults who wish for death. Clin Gerontol. (2020) 43:4–16. doi: 10.1080/07317115.2019.1672846, PMID: 31615349

[20] Chang Q Chan CH Yip PSF. A meta-analytic review on social relationships and suicidal ideation among older adults. Soc Sci Med. (2017) 191:65–76. doi: 10.1016/j.socscimed.2017.09.003, PMID: 28910599

[21] Chu C Buchman-Schmitt JM Stanley IH Hom MA Tucker RP Hagan CR. The interpersonal theory of suicide: a systematic review and meta-analysis of a decade of cross-national research. Psychol Bull. (2017) 143:1313–45. doi: 10.1037/bul0000123 29072480 PMC5730496

[22] Joiner TE van Orden KA Witte TK Selby EA Ribeiro JD Lewis R. Main predictions of the interpersonal-psychological theory of suicidal behavior. J Abnorm Psychol. (2009) 118:634–46. doi: 10.1037/a0016500, PMID: 19685959 PMC2846517

[23] Inglis T. Church and culture in Catholic Ireland. Studies. (2017) 106:21–30. doi: 10.1353/stu.2017.0015

[24] Central Statistics Office. Census of population summary tables (2023). Available at: https://data.cso.ie (Accessed October 2023).

[25] Krause N. Assessing the relationships among religiousness, loneliness, and health. Arch Psychol Relig. (2016) 38:278–300. doi: 10.1163/15736121-12341330

[26] Orr J Tobin K Carey D Kenny RA McGarrigle C. Religious attendance, religious importance, and the pathways to depressive symptoms in men and women aged 50 and over living in Ireland. Res Aging. (2019) 41:891–911. doi: 10.1177/0164027519860270 31331248

[27] Huang L Tsai Y Liu C Chen YJ. Influencing and protective factors of suicidal ideation among older adults. Int J Mental Health Nurs. (2017) 26:191–9. doi: 10.1111/inm.12247 27452945

[28] Dine RL. You shall bury him: burial, suicide and the development of Catholic law and theology. Med Humanit. (2020) 46:299–310. doi: 10.1136/medhum-2018-011622 31350305

[29] Donoghue O McGarrigle C Foley M Fagan A Meaney J Kenny RA. Cohort profile update: the Irish longitudinal study on ageing (TILDA). Int J Epidemiol. (2018) 47:1398–1398l. doi: 10.1093/ije/dyy163, PMID: 30124849

[30] Kearney PM Cronin H O'Regan C Kamiya Y Savva GM Whelan B. Cohort profile: the Irish longitudinal study on ageing. Int J Epidemiol. (2011) 40:877–84. doi: 10.1093/ije/dyr116, PMID: 21810894

[31] Kenny RA Whelan B Cronin H. *The Design of the Irish Longitudinal Study on ageing.* The Irish Longitudinal Study on Ageing (2010).

[32] Whelan BJ Savva GM. Design and methodology of the Irish longitudinal study on ageing. J Am Geriatr Soc. (2013) 61:265–8. doi: 10.1111/jgs.12199 23662718

[33] Russell D. The UCLA loneliness scale (version 3): reliability, validity, and factor structure. J Pers Assess. (1996) 66:20–40. doi: 10.1207/s15327752jpa6601_2, PMID: 8576833

[34] Radloff LS. A self-report depression scale for research in the general population. Appl Psychol Meas. (1977) 1:385–401. doi: 10.1177/014662167700100306

[35] O’Halloran AM Kenny RA King-Kallimanis BL. The latent factors of depression from the short forms of the CES-D are consistent, reliable and valid in community-living older adults. Eur Geriatr Med. (2014) 5:97–102. doi: 10.1016/j.eurger.2013.12.004

[36] Vilagut G Forero CG Barbaglia G Alonso J. Screening for depression in the general population with the Center for Epidemiologic Studies Depression (CES-D): a systematic review with meta-analysis. PLoS ONE. (2016) 11:e0155431. doi: 10.1371/journal.pone.0155431 27182821 PMC4868329

[37] StataCorp. Stata Statistical Software: Release 15 (2017).

[38] R Core Team. R: A Language and Environment for Statistical Computing (2023).

[39] Jayasekara R Procter N Harrison J Skelton K Hampel S Draper R. Cognitive behavioural therapy for older adults with depression: a review. J Ment Health. (2015) 24:168–71. doi: 10.3109/09638237.2014.971143 25358075

[40] Hagan R Manktelow R Taylor BJ Mallett J. Reducing loneliness amongst older people: a systematic search and narrative review. Aging Ment Health. (2014) 18:683–93. doi: 10.1080/13607863.2013.875122 24437736

[41] Nurminen M Thoma-Otremba A Casabianca E. *Mapping of loneliness interventions in the EU*. European Commission (2023).

[42] Smith R Wuthrich V Johnco C Belcher J. Effect of group cognitive behavioural therapy on loneliness in a community sample of older adults: a secondary analysis of a randomized controlled trial. Clin Gerontol. (2021) 44:439–49. doi: 10.1080/07317115.2020.1836105 33100187

[43] Kubzansky LD Epel ES Davidson RJ. Prosociality should be a public health priority. Nat Hum Behav. (2023) 7:2051–3. doi: 10.1038/s41562-023-01717-3, PMID: 37857873 PMC10840689

[44] Ward M Briggs R McGarrigle CA de Looze C O’Halloran AM Kenny RA. The bi-directional association between loneliness and depression among older adults from before to during the COVID-19 pandemic. Int J Geriatr Psychiatry. (2023) 38:e5856. doi: 10.1002/gps.5856, PMID: 36462183

[45] Ganiel G. Online opportunities in secularizing societies? Clergy and the COVID-19 pandemic in Ireland. Religions. (2021) 12:437. doi: 10.3390/rel12060437

