# Supplementary material for: Social disconnection correlates of a “Wish to Die” among a large community-dwelling cohort of older adults
Source: Front Public Health. 2024 Aug 21;12:1436218. doi: 10.3389/fpubh.2024.1436218 (PMC11371793; doi:10.3389/fpubh.2024.1436218)
Supplement: Supplementary file 1 [file Table_1.DOCX]

**Table S1.** Prevalence of multiple indicators of social disconnection among older adults expressing a WTD (CAPI participants, N=8,171)

|  | **All** | **No WTD** | **Yes WTD** | **Sig. Test** |
| --- | --- | --- | --- | --- |
|  | **% (95% CI)** | **% (95% CI)** | **% (95% CI)** |  |
| Lives alone | 22.9 (21.8,24.0) | 94.5 (93.3,95.5) | 5.5 (4.5,6.7) | χ2(1) = 24.13,  p<0.001 |
| Lives with others | 77.1 (76.0,78.2) | 96.9 (96.4,97.4) | 3.1 (2.6,3.6) |  |
| Not married | 32.2 (30.9,33.5) | 94.8 (93.8,95.7) | 5.2 (4.3,6.2) | χ2(1) = 26.48,  p<0.001 |
| Married | 67.8 (66.5,69.1) | 97.1 (96.6,97.6) | 2.9 (2.4,3.4) |  |
| No social participation | 55.0 (53.6,56.4) | 95.5 (94.7,96.1) | 4.5 (3.9,5.3) | χ2(1) = 23.87,  p<0.001 |
| Yes social participation | 45.0 (43.6,46.4) | 97.5 (96.9,98.0) | 2.5 (2.0,3.1) |  |
| Volunteer | 50.3 (48.8,51.7) | 97.6 (97.0,98.1) | 2.4 (1.9,3.0) | χ2(1) = 18.70,  p<0.001 |
| No volunteer | 49.7 (48.3,51.2) | 95.7 (94.9,96.4) | 4.3 (3.6,5.1) |  |
| Frequency attends church | |  |  |  |
| Never | 15.7 (14.5,17.0) | 92.0 (90.2,93.4) | 8.0 (6.6,9.8) | χ2(2) = 90.50,  p<0.001 |
| Monthly | 26.4 (25.3,27.6) | 96.5 (95.7,97.3) | 3.5 (2.7,4.3) |  |
| Weekly | 57.9 (56.2,59.5) | 97.5 (97.0,98.0) | 2.5 (2.0,3.0) |  |

**Table S2.** Results of logistic regressions to estimate the association between social disconnection indicators and WTD, controlling for socio-demographic characteristics (CAPI participants, N=8,171).

|  | **Lives**  **alone** | **Married** | **Social participation** | **Attends church** |
| --- | --- | --- | --- | --- |
|  | **O.R. (95% CI)** | **O.R. (95% CI)** | **O.R. (95% CI)** | **O.R. (95% CI)** |
| Unadjusted | 1.84  (1.41,2.38)*** | 1.83  (1.43,2.35)*** | 1.85 (1.43,2.40)*** | 3.06 (2.36,3.98)*** |
| Adjusted | 1.51  (1.12,2.03)** | 1.33  (1.00,1.78)* | 1.26  (0.94,1.68) | 2.29 (1.69,3.08)*** |
| Female | 1.02  (0.79,1.32) | 0.99  (0.76,1.28) | 0.99  (0.77,1.29) | 1.08  (0.83,1.41) |
| Age | 0.98  (0.97,1.00)* | 0.98  (0.97,1.00) | 0.99  (0.97,1.00) | 0.99  (0.98,1.01) |
| Secondary | 0.69  (0.51,0.94)* | 0.70  (0.51,0.95)* | 0.71  (0.52,0.97)* | 0.71  (0.52,0.97)* |
| 3rd level | 0.60  (0.41,0.88)** | 0.61  (0.42,0.89)* | 0.65  (0.44,0.96)* | 0.59 (0.40,0.87)** |
| Rural | 0.72  (0.55,0.95)** | 0.72  (0.55,0.96)* | 0.72  (0.54,0.95)* | 0.81  (0.61,1.07) |
| Depressed | 15.46 (11.57,20.66)*** | 15.52 (11.56,20.84)*** | 15.85 (11.89,21.13)*** | 15.20 (11.40,20.27)*** |

*p<0.05, **p<0.01, ***p<0.001
